# Supplementary material for: ThirdPeak is a flexible tool designed for the robust analysis of two- and three-dimensional tracking data
Source: Commun Biol. 2024 Dec 20;7:1683. doi: 10.1038/s42003-024-07378-w (PMC11659616; doi:10.1038/s42003-024-07378-w)
Supplement: Supplementary file 1 — Supplementary Information [file 42003_2024_7378_MOESM1_ESM.pdf]

Supplementary figures

	ThirdPeak[1]	SMAP[2]	Swift[3]	TrackMate[4]	u-Track 3D[5]	TrackIt[6]	Decode[7]	Picasso[8]	NOBIAS[9]	SMAUG[10]	ExTrack[11]	Tardis[12]	anaDD[13]	SPTAnalysis[14]
Graphical interface	✓	✓	✓	✓	(✓)	✓	X	✓	X	X	(✓)	✓	X	✓
2D Analysis	✓	✓	✓	✓	✓	✓	✓	✓	✓	✓	✓	✓	✓	✓
3D Analysis	✓	✓	✓	(✓)	(✓)	X	✓	✓	X	X	✓	X	X	X
Batch processing	✓	✓	✓	(✓)	(✓)	✓	(✓)	X	(✓)	(✓)	(✓)	✓	(✓)	(✓)
CSV import	✓	X	✓	✓	X	✓			X	X	X	✓	X	✓
Localizing	(✓)	✓		✓	✓	✓	✓	✓						
Tracking	(✓)		✓	✓	✓	✓			✓	✓	✓	✓	✓	
Validating	✓			✓	✓	✓		✓	X	X	X	X	X	(✓)
Analysing	✓		(✓)	✓	✓	✓						✓		✓
Generating Supermaps	✓			X	X	X								✓
Open source	✓	✓	✓	✓	X*	X*	✓	✓	X*	X*	✓	✓	X*	✓

✓ = implemented (✓) = partly implemented, additional software or scripting required

X = not implemented X* = MATLAB licence necessary ■ = not in scope of the software

Supplementary figure 1: Overview of software used for single molecule localization and general tracking. All the packages are provided in a format that allows them to be set up by a user without in-depth programming knowledge. Full citations see [1–14]

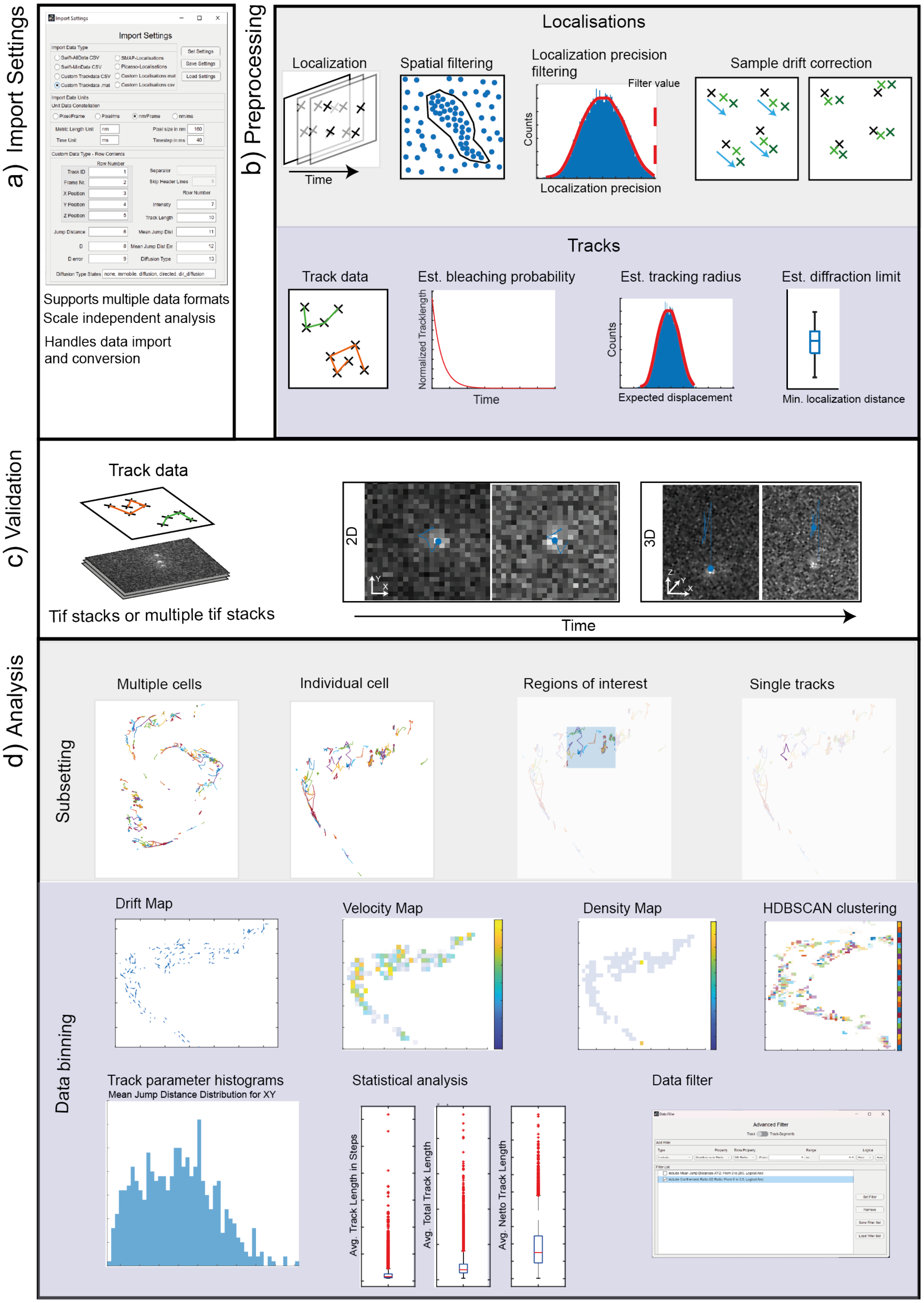

Supplementary figure 2: Feature overview of ThirdPeak on an exemplaray 3D data set of surface diffusion on *Trypanosoma brucei*. a) The import dialogue starts the chosen workflow (preprocessing, validation or analaysis) to determine the expected data format. **b)** During preprocessing, localisation data can be filtered on their spatial position, precision values or

intensity. Next, histograms of the localisations are generated and saved in the current working directory. An automatic drift correction can be applied if desired, either using a mean shift approach or fiducials. The processed localisations can then be tracked if swift is installed on the computer directly from the GUI of ThirdPeak. Alternatively, experimentally generated track data can be loaded into the preprocessing workflow to determine bleach probability, diffraction limit and the expected displacement value to refine the tracking process. If sufficient data is present, the fourier ring correlation or fourier shell correlation can be calcualted to determine the resolution of the resolved structure. If before mentioned methods fail to produce a resolution estimation due to insufficient data, the diffraction limit can be determined by the minimal distance of the localisation per frame. After each step, a file will be generated that allows the user to return to a given step, or altogether switch to a different analysis software. **c)** During the validation step, localisation or track data can be used with the original tif images to validate and, if necessary, refine the localisation and tracking results. Both timeseries of single tif images or tif stacks are supported. **d)** The analysis workfow allows visualisation of the track data, subsetting it into single tracks or region of interest. Alternatively, the data can be binned to visualize locally dominating dynamics, either by their overall drift, their velocities or by externally calculated data from the tracking algorithm or DeepSPT if it is available. Automated clustering using the HDBSCAN approach is also possible. Data can then be visualized in histograms or used for statistical analysis. The track data can be further filtered using the calculated track properties.

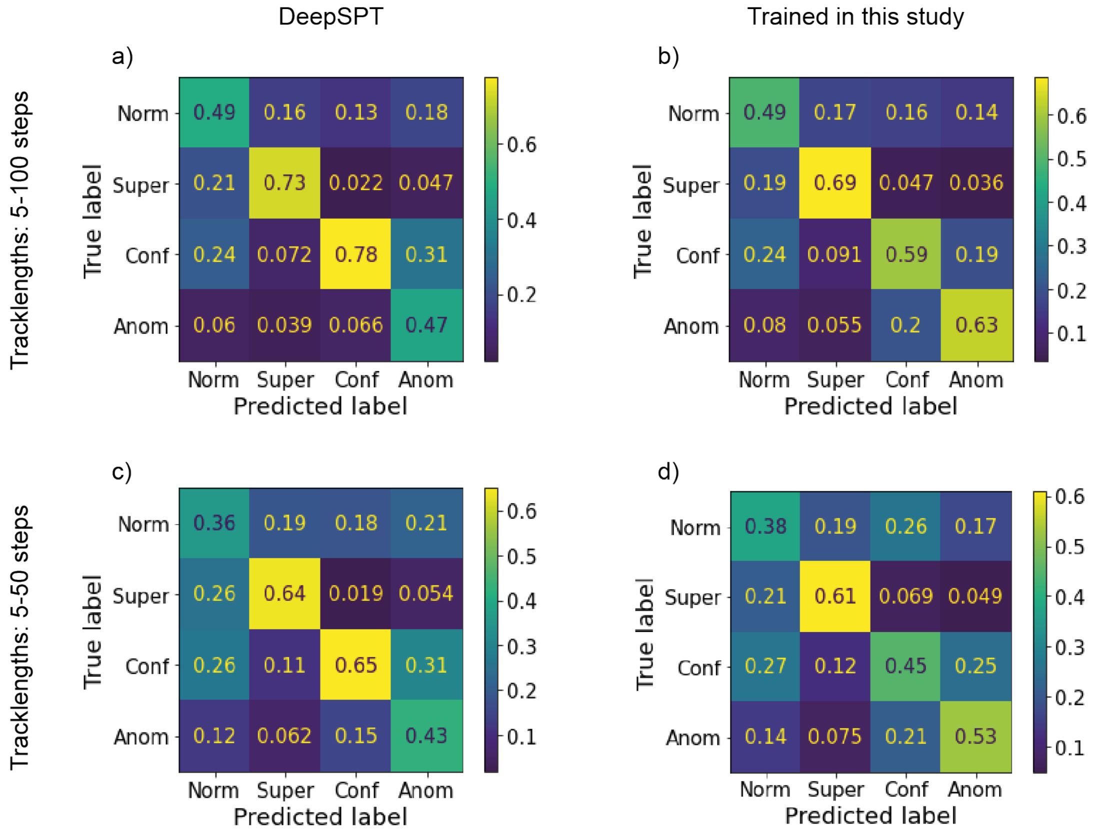

Supplementary figure 3: Confusion matrices of the temporal segmentation network for 3D tracks using the provdied and the self-trained network . a) and **b)** show the confusion matices of classifications made for test tracks consisting of 5-100 steps for both the provided (DeepSPT) and the self-trained network. Both network show an nearly equal percentage of correct classification for the four respective diffusion types (Norm: Normal, Brownian motion; Super: Superdiffusive behaviour; Conf: Confied motion; Anom: Anomalous diffusion). While the provided network is better in classfiying confined motion, the self-trained network achieves better results for anomalous diffusion. **c)** and d) show the confusion matrices for test data consting of shorter tracks (5-50 steps). With less information, the networks classify less tracks correctly. They still perform better than chance (0.25).

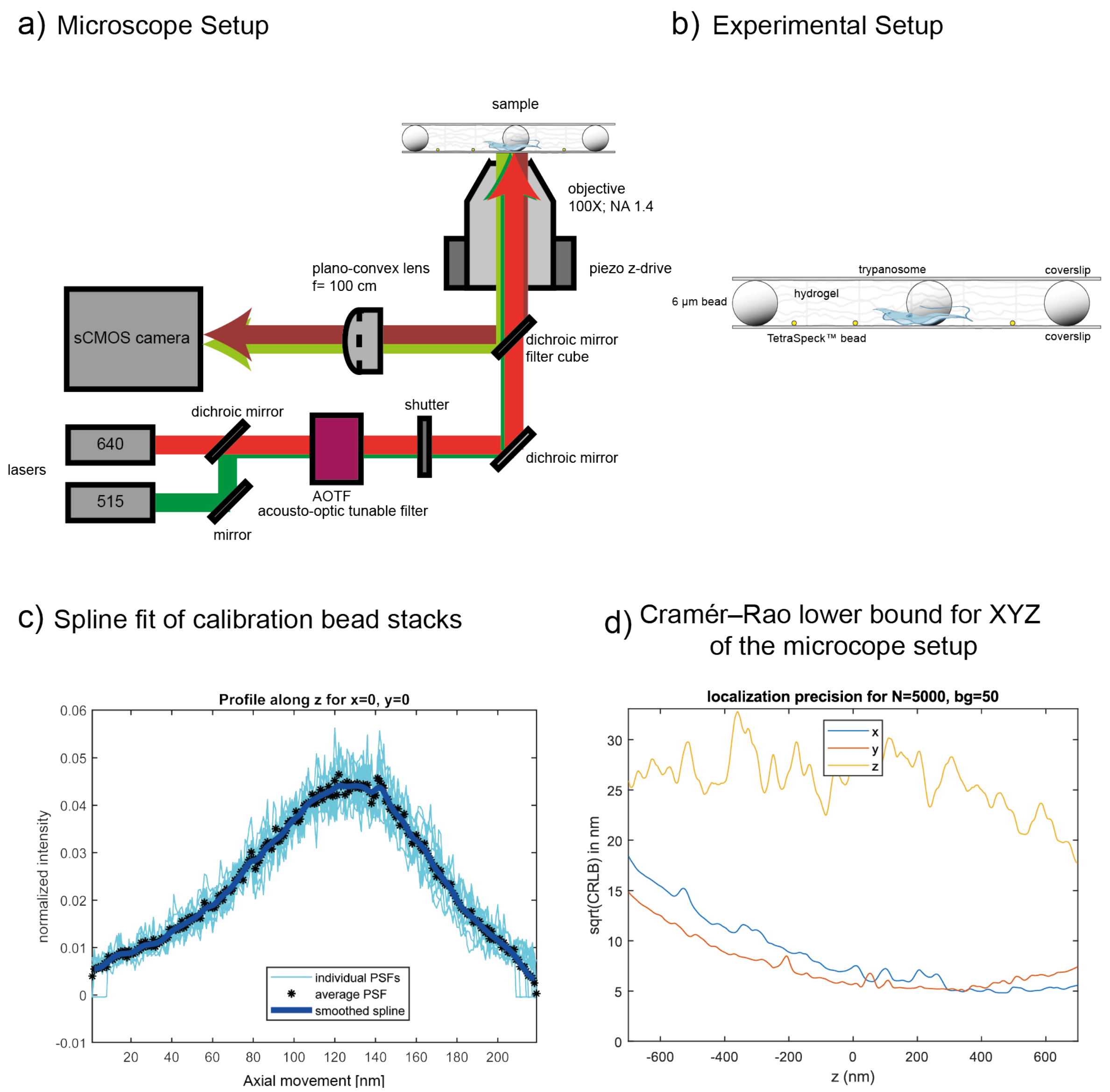

Supplementary figure 4: Overview of the microscope and experimental setup used as well as the associated 3D calibration and Cramer-Rao lower bound. a) For single molecule imaging, an inverted widefield microscope (Leica DMI6000B) with two laser lines at 515 nm and 640 nm is used. The protein of interest is labelled with 0.5-1 nM ATTO-643-NHS (ATTO-TEC) and excited by the 640 nm laser. The cytosol is labelled with 10 nM orgeon green (Promega) for finding and focusing on the cells. The laser line can be rapidly switched using an AOTF. The emitted light is then directed through the astigmatic lens to encode the z position of the emitters by the shape of their point spread function. **b)** To image living, immobilized cells we use a hydrogel based on polyethyleneglycole and hyaluronic acid[15]. Spacer beads allow us to define the sample hight to six micrometer and TetraSpeck beads in the sample can be used to determine the drift. **c)** For the determination of the z position of single emitters, z-stacks of fluorescent TetraSpeck beads are acquired. **d)** With these image stacks, a calibration curve can be calculated with SMAP and the Cramer-Rao lower bound can be determined. This value describes the therotically best possible precision for the given dimension. Due to the astigmatism and the associated z-dependent elongation of the point spread function in the lateral plane, it becomes more difficult to determine the true lateral positions, resulting in reduced

precision at the lower and upper limit of the calibrated range. For the axial position, the theoretically best resolution is usually around 35-40 nm in the calibrated range.

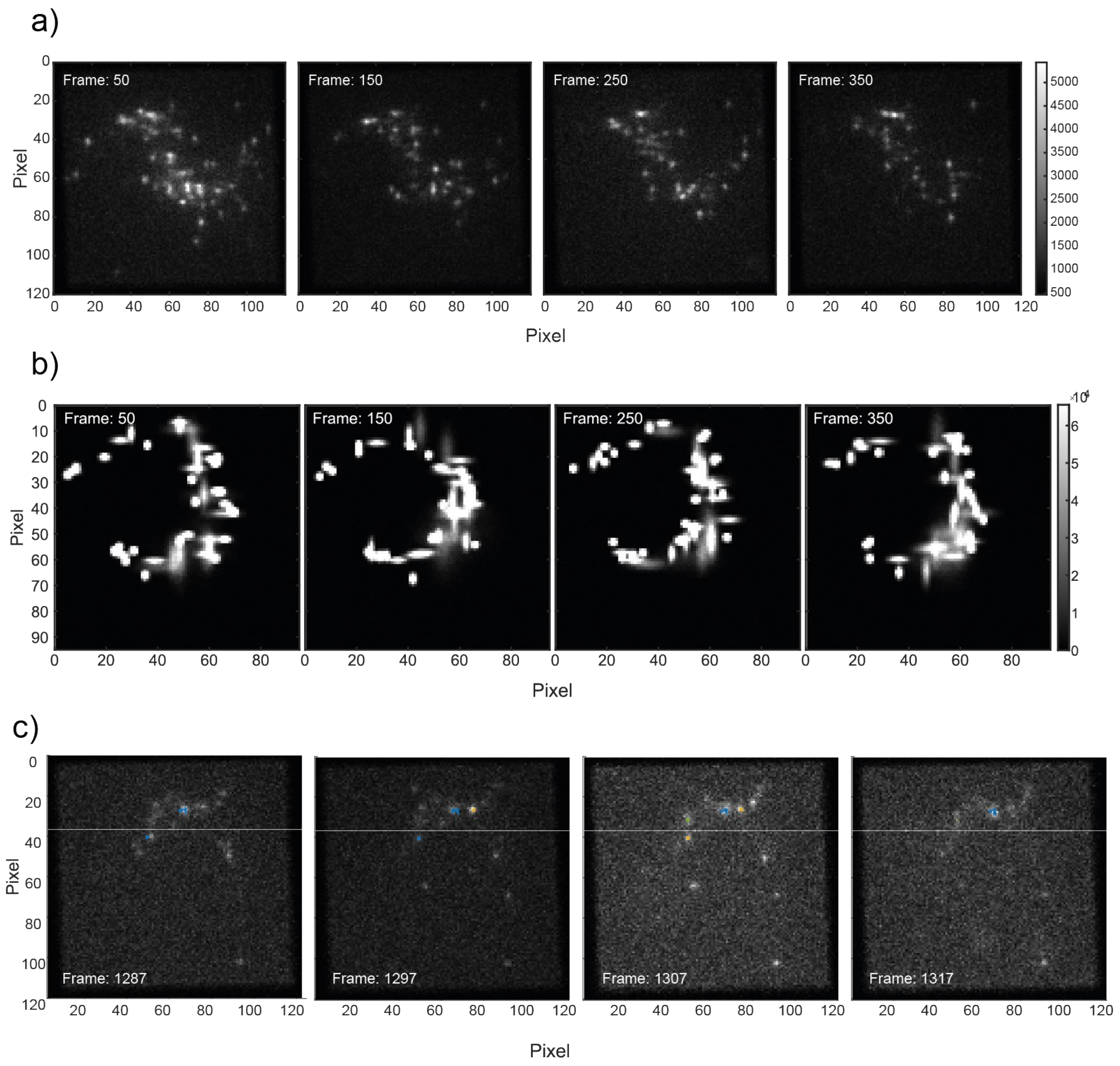

Supplementary figure 5: Images from the experiment, the simulation and the validation of the tracking process on experimental data. a) Four exemplary images of immobilized Trypanosoma brucei labelled with A643-NHS. The cells were imaged at 3 kW/cm^2 for 2500 frames. The maximum intensity of the emitters were around 6000 a.u. **b)** Four exemplary images of the simulated immter data generated using SMIS2.1. The overall intesity was ten times greater than the experimental data and no noise was added. **c)** Validation overlay with the localisations (spots) and tracks (lines) of exemplary images from the experiments. The pixel size is 160 nm for all images, experimentally and simulated.

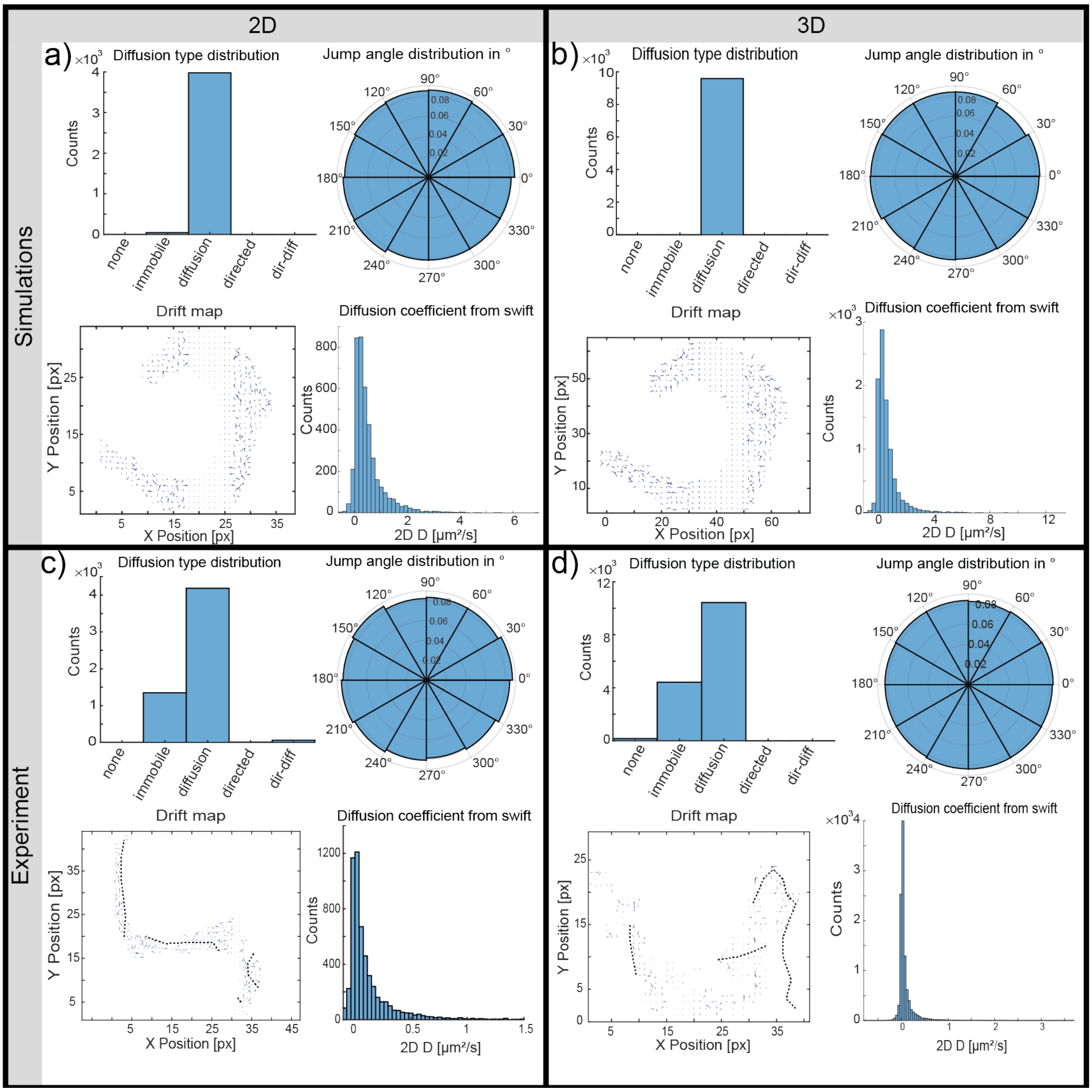

Supplementary figure 6: Additional track properties of simulated and experimental diffusion data. The simulated data shows that all tracked particles are diffusive, while for the experimental data, a smaller fraction of 20-30% is considered immobile by the swift algorithm. A particle is considered immobile if it is not moving more than the value determined by the localisation precision. The jump angle distribution shows an even distribution for all conditions. A primary jump angle around 0° points to directed transport, while a jump angle of 180° indicates a largr amount of immobile particles. The drift map illustrates the overall direction of the tracks on their given position in the grid. For the simulations, neither sample drift nor directionality onto a given position is visible. In the center part of the simulated area, the drift vectors are small, coinciding with the small velocity values, which is probably a result of the model used. For the experimental data, drift vectors are often parallel to the flagella, as particles can not pass to the flagellar membrane. Drift vectors all pointing to the center of a circle can indicate the flagellar pocket of the cell, which is not present in the simulated data. The dotted black line marks the location of the flagellum.. The diffusion coefficient determined by swift does not show a large difference between the data sets. It has to be noted that this diffusion coefficient calculated by swift is only determined in 2D. This also applies for the 3D data.

Supplementary table 1: Results of the track analysis

Property	Simulation 2D	Simulation 3D	Experiment 2D	Experiment 3D
Avg. Number of steps (Min, Median, Max)	5, 14, 729	5,11,130	5, 7, 494	5, 8, 330
Total Track Length (Min, Median, Max)	272, 3508, 101310	315, 3529, 48329	85, 990, 33955	1, 7, 221
Net Track Length (Min, Median, Max)	17, 3508, 5904	3, 760, 5361	1, 238, 2497	0, 1, 14
Diffusion coefficient by cumulative jump distance (R^2, Effective D, D1+-S.E.M, D2+- S.E.M) $\mu m^2/s$	1, 454, 653±21, 97±7	1, 700, 903±40, 114±15	1, 303, 501±10, 95±2	1, 471, 1120±35, 79±1
Mean jump distance distribution	225, 170<br>387, 150	200, 98<br>348, 168<br>508, 267	129, 96<br>215, 145	79, 49<br>125, 85<br>191, 141

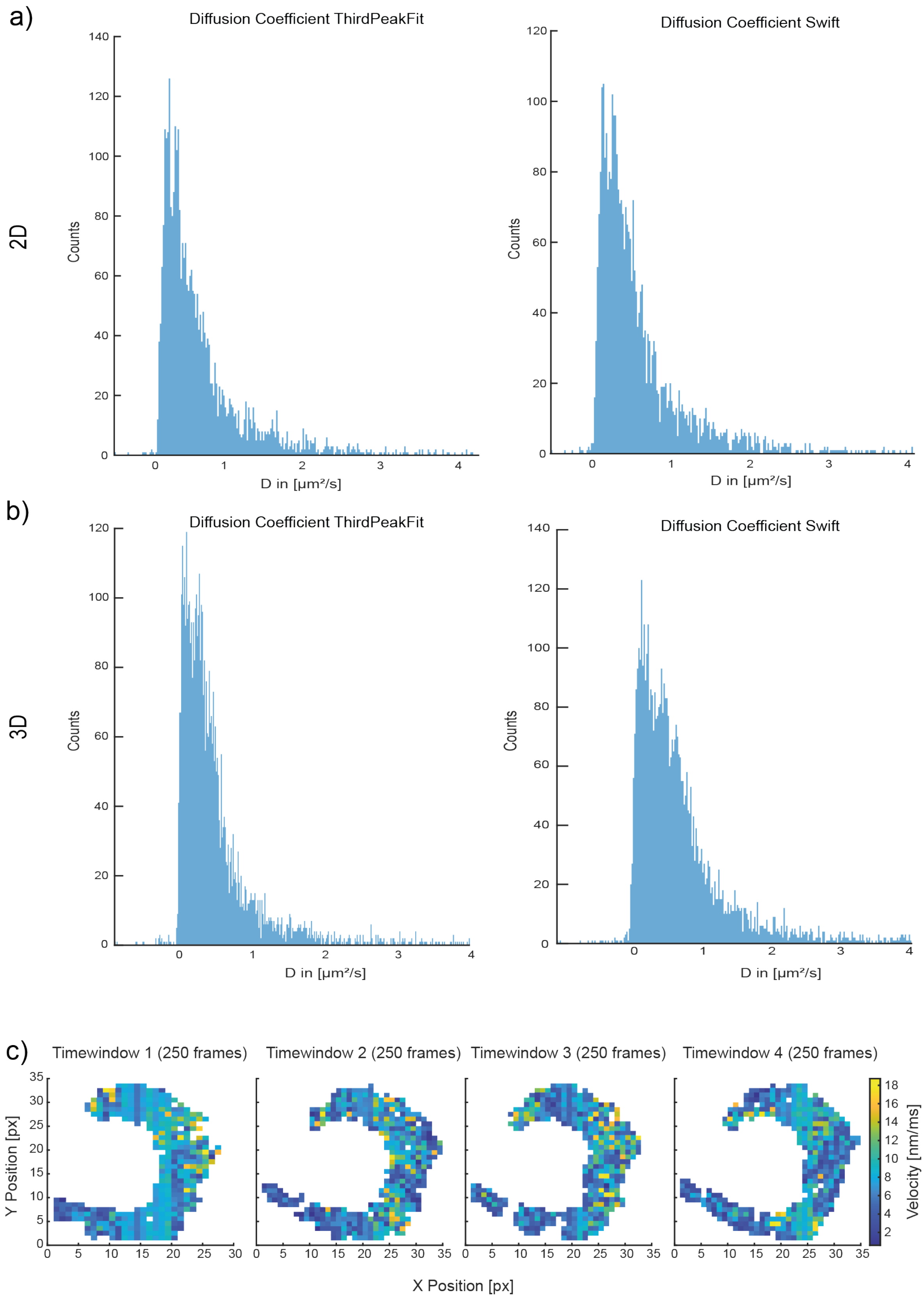

Supplementary figure 7: Comparison of the calculated diffusion coefficients using the mean squared displacement approach from ThirdPeak and swift in 2D and 3D and a

timeseperated heatmap showing local diffusion differences in the simulated data. a, b) Comparison of the calculated diffusion coefficient of ThirdPeak and swift on the two-dimensional **(a)** and three-dimensional **(b)** simulated data. Both histograms look nearly identical. Two very prominent peaks are visibile for one value slightly below 0.5 µm²/s and above 0.5 µm²/s, as well as an additional smaller fraction around 1 µm²/s, at which the histograms flattens into a righward shoulder. These values are in good agreement with the parameters set for the simulation (0.16 µm²/s, 0.56 µm²/s and 1.33 µm²/s). **c)** Splitting the velocity values of the simulated data into time windows each containing 250 frames allos to identify temporally and locally differences in the data that are otherwise averaged out by the slow diffusing population. The pixel size is 320 nm.

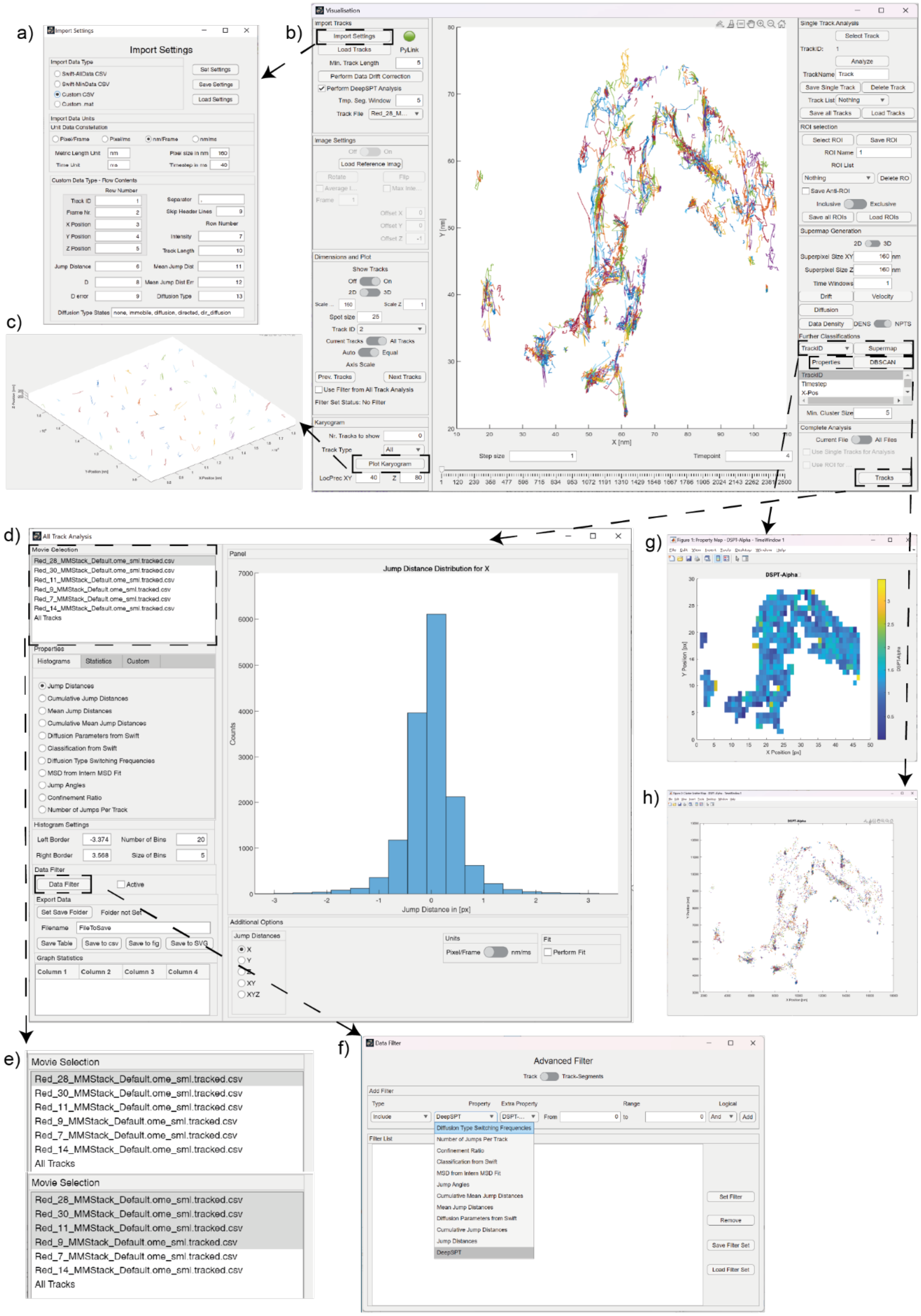

Supplementary figure 8 : Screenshots of ThirdPeak. a) Data import dialogue which allows to describe the data format that will be importet and the scaling of the data. b) General overview of the visualisation and analysis workflow interface. c) Overview of the tracks, lined up in a grid. d) Track analysis window right after opening it. e) Data selection options.Either a

single file, multiple files or all files can be selected. f) Options of the advanced filter dialogue in more detail. g) Heatmap generated by data from DeepSPT. h) Cluster analysis using the HDBSCAN algorithm.

References

1. Müller, T. ThirdPeak: A flexible tool designed for the robust analysis of two- and three-dimensional (single-molecule) tracking data [Dataset]. (Zenodo, 2024).

2. Ries, J. SMAP: a modular super-resolution microscopy analysis platform for SMLM data. *Nat Methods* **17**, 870–872 (2020).

3. Endesfelder, M., Schießl, C., Turkowyd, B., Lechner, T. & Endesfelder, U. swift – fast, probabilistic tracking for dense, highly dynamic single-molecule data. *manuscript in prep.*

4. Ershov, D. *et al.* TrackMate 7: integrating state-of-the-art segmentation algorithms into tracking pipelines. *Nat. Methods* **19**, 829–832 (2022).

5. Roudot, P. *et al.* u-track3D: Measuring, navigating, and validating dense particle trajectories in three dimensions. *Cell Rep. Methods* 100655 (2023) doi:10.1016/j.crmeth.2023.100655.

6. Kuhn, T., Hettich, J., Davtyan, R. & Gebhardt, J. C. M. Single molecule tracking and analysis framework including theory-predicted parameter settings. *Sci Rep-uk* **11**, 9465 (2021).

7. Speiser, A. *et al.* Deep learning enables fast and dense single-molecule localization with high accuracy. *Nat Methods* **18**, 1082–1090 (2021).

8. Schnitzbauer, J., Strauss, M. T., Schlichthaerle, T., Schueder, F. & Jungmann, R. Super-resolution microscopy with DNA-PAINT. *Nat Protoc* **12**, 1198–1228 (2017).

9. Chen, Z., Geffroy, L. & Biteen, J. S. NOBIAS: Analyzing Anomalous Diffusion in Single-Molecule Tracks With Nonparametric Bayesian Inference. *Frontiers Bioinform* **1**, 742073 (2021).

10. Karslake, J. D. *et al.* SMAUG: Analyzing single-molecule tracks with nonparametric Bayesian statistics. *Methods* **193**, 16–26 (2021).

11. Simon, F., Tinevez, J.-Y. & Teeffelen, S. van. ExTrack characterizes transition kinetics and diffusion in noisy single-particle tracks. *J Cell Biol* **222**, e202208059 (2023).

12. Martens, K. J. A., Turkowyd, B., Hohlbein, J. & Endesfelder, U. Temporal analysis of relative distances (TARDIS) is a robust, parameter-free alternative to single-particle tracking. *Nat. Methods* 1–8 (2024) doi:10.1038/s41592-023-02149-7.

13. Vink, J. N. A., Brouns, S. J. J. & Hohlbein, J. Extracting Transition Rates in Particle Tracking Using Analytical Diffusion Distribution Analysis. *Biophys. J.* **119**, 1970–1983 (2020).

14. Parutto, P. *et al.* High-throughput super-resolution single-particle trajectory analysis reconstructs organelle dynamics and membrane reorganization. *Cell Reports Methods* **2**, 100277 (2022).

15. Schwebs, M. *et al.* Single-molecule fluorescence microscopy demonstrates fast dynamics of the variant surface glycoprotein coat on living trypanosomes. *Biorxiv* 2022.08.03.502583 (2022) doi:10.1101/2022.08.03.502583.
